# Healthcare-Associated Infections in Critically Ill COVID-19 Patients Across Evolving Pandemic Waves: A Retrospective ICU Study †

**DOI:** 10.3390/medicina62010118

**Published:** 2026-01-06

**Authors:** Nihan Altintepe Baskurt, Esra Akdas Tekin, Onur Okur, Namigar Turgut

**Affiliations:** Department of Anesthesiology and Intensive Care, Prof. Dr. Cemil Tascioglu State Hospital, 34384 Istanbul, Turkey; dr_esraktas@yahoo.com (E.A.T.); onurokur@live.com (O.O.); namigarturgut@gmail.com (N.T.)

**Keywords:** antibiotic susceptibility profile, antimicrobial resistance, COVID-19, healthcare-associated infections, intensive care, multidrug-resistant organisms, nosocomial infections, pandemics

## Abstract

*Background and Objectives*: Healthcare-associated infections (HAIs) significantly increase morbidity and mortality in critically ill patients, and their burden became more pronounced during the COVID-19 pandemic. However, data describing the temporal evolution of HAIs, pathogen distribution, and associated risk factors across consecutive pandemic waves remain limited. This study aimed to characterize the epidemiology, microbiology, and outcomes of HAIs in COVID-19 intensive care units (ICU) patients and to identify clinical and laboratory predictors of mortality. *Materials and Methods*: This retrospective observational study included adult patients with RT-PCR–confirmed COVID-19 who developed at least one HAI ≥ 48 h after ICU admission between March 2020 and December 2020, encompassing the first three pandemic waves in Türkiye, in a tertiary-care ICU. Demographic, clinical, laboratory, and microbiological data were collected. Inflammatory markers and severity scores (SAPS-II, MCCI, and NLR) were analyzed. Receiver operating characteristic (ROC) curve analysis was used to determine optimal cut-off values for mortality prediction. *Results*: Among the 1656 ICU admissions, 145 patients (8.7%) developed HAIs; after exclusions, 136 patients were included in the final analysis. Bloodstream infections were the most frequent HAI (57%), followed by urinary tract infections (31%), ventilator-associated pneumonia (9%), and surgical site infections (1%). *Klebsiella pneumoniae* was the predominant pathogen, followed by *Candida albicans* and *Acinetobacter baumannii*. Multidrug-resistant organisms, including MRSA and VRE, showed variable distribution across pandemic periods. Overall in-hospital mortality was 74.3%. Non-survivors had significantly higher SAPS-II, MCCI, and NLR values. ROC analysis identified NLR > 38.8 and SAPS-II > 35.5 as mortality-predictive thresholds. Dynamic inflammatory marker patterns correlated with infection timing, and early peaks of CRP, WBC, and IL-6 were associated with worse outcomes. *Conclusions*: HAIs imposed a substantial clinical burden on critically ill COVID-19 patients, with high mortality driven predominantly by multidrug-resistant bloodstream infections. Severity indices and inflammation-based biomarkers demonstrated strong prognostic value. Temporal shifts in pathogen ecology across pandemic waves underscore the need for adaptive infection-prevention strategies, continuous microbiological surveillance, and strengthened antimicrobial stewardship in critical care settings.

## 1. Introduction

Healthcare-associated infections (HAIs) remain a major source of morbidity, mortality, and healthcare expenditure worldwide, with intensive care units (ICUs) bearing a disproportionate burden due to invasive procedures, device utilization, and high illness severity [1]. The COVID-19 pandemic placed unprecedented strain on ICU infrastructure, profoundly altering patient flow, staffing patterns, infection prevention practices, and antimicrobial use, thereby reshaping the epidemiology of ICU-acquired infections [2,3,4].

During successive pandemic surges, reports from different healthcare systems described divergent trends in HAIs [5]. While enhanced infection-control measures in some centers were associated with reductions in selected HAIs, others reported substantial increases in bloodstream infections and ventilator-associated pneumonia, particularly during periods of high device utilization and workforce strain [6,7]. These heterogeneous findings suggest that the impact of COVID-19 on HAI epidemiology is highly context dependent and influenced by local healthcare capacity and resource constraints.

Secondary bacterial and fungal infections have emerged as key determinants of adverse outcomes in critically ill COVID-19 patients. Multidrug-resistant (MDR) pathogens—most notably *Klebsiella pneumoniae*, *Acinetobacter baumannii*, methicillin-resistant *Staphylococcus aureus* (MRSA), and vancomycin-resistant enterococci (VRE)—have been disproportionately reported in COVID-19 ICUs and are associated with markedly increased mortality, especially in patients requiring prolonged mechanical ventilation [3,8,9]. The convergence of viral-induced immune dysregulation, extensive antimicrobial exposure, and sustained invasive support creates a clinical environment highly conducive to nosocomial infection and antimicrobial resistance [5,9,10,11,12].

Despite expanding literature, data remain limited from middle-income countries, including Türkiye, where ICU capacity, antimicrobial resistance patterns, and pandemic-related system pressures differ from those in high-income settings. Moreover, few studies have examined temporal changes in HAI epidemiology, pathogen distribution, and mortality risk across distinct pandemic waves within a defined ICU population [5,11,13]. The dynamic relationship between inflammatory burden, infection timing, and outcomes in this setting also remains incompletely characterized.

Therefore, this study aimed to evaluate the epidemiology, microbiological characteristics, antimicrobial resistance patterns, and clinical predictors of mortality among ICU patients with COVID-19 who developed HAIs during the first three pandemic waves in a tertiary teaching hospital in Istanbul, Türkiye. By integrating temporal comparisons with severity and inflammation-based risk assessment, this study seeks to provide clinically relevant insights to inform infection prevention strategies and antimicrobial stewardship in high-acuity ICU settings during current and future public health crises.

## 2. Materials and Methods

### 2.1. Study Design and Setting

This retrospective observational study was conducted in the adult ICU of a tertiary-care teaching hospital in Istanbul, Türkiye. The ICU consists of 60 beds organized as mixed medical–surgical units and is managed by board-certified intensivists.

The study period extended from 31 March 2020 to 31 December 2020, encompassing the first three waves of the COVID-19 pandemic in Türkiye, as defined by national epidemiological data and ICU admission peaks. This nine-month period was divided into three consecutive quarterly phases to allow for longitudinal comparison: April–June 2020 (first period), July–September 2020 (second period), and October–December 2020 (third period).

Ethical approval was obtained from the Prof. Dr. Cemil Taşçıoğlu City Hospital Clinical Research Ethics Committee (Approval No.: 173; Date: 19 April 2021). The requirement for informed consent was waived due to the retrospective nature of the study.

### 2.2. Patient Selection

Eligible participants included adult patients (≥18 years) who were admitted to the ICU with RT-PCR–confirmed SARS-CoV-2 infection, developed at least one HAI ≥ 48 h after ICU admission, and had complete clinical, laboratory, and microbiological documentation.

Patients were excluded if they had community-acquired infections on admission, prior documented COVID-19 infection within the past 90 days, incomplete medical records, or transfer to another facility before HAI evaluation.

HAIs were defined using the Centers for Disease Control and Prevention (CDC) National Healthcare Safety Network (NHSN) criteria [14]. COVID-19 disease severity was assessed and verified by infectious disease specialists and intensivists following national guidelines.

Because microbiological surveillance from non-ICU hospital wards was not included, the findings primarily reflect the epidemiology of this specific ICU and may limit generalizability.

### 2.3. Data Collection and Clinical Parameters

Demographic characteristics, comorbidities, invasive device exposure, laboratory parameters, infection characteristics, and clinical outcomes were extracted from the institutional electronic medical record system using a standardized form.

Disease severity at ICU admission was assessed using the Charlson Comorbidity Index (CCI), Simplified Acute Physiology Score II (SAPS II), and Neutrophil-to-Lymphocyte Ratio (NLR). Patients were categorized into three temporal groups corresponding to the predefined pandemic periods to enable longitudinal comparisons.

Inflammatory markers—including C-reactive protein (CRP), white blood cell (WBC) count, interleukin-6 (IL-6), NLR, and platelet-to-lymphocyte ratio (PLR)—were evaluated both as isolated measurements and dynamically. Peak values and temporal relationships with the timing of microbiological culture positivity were analyzed. Correlation analyses were performed to assess associations between inflammatory marker kinetics, time to first pathogen isolation, and clinical outcomes.

### 2.4. Microbiological Analysis

Blood, urine, respiratory samples, and catheter tips were processed in the hospital’s microbiology laboratory. Microorganism identification and antimicrobial susceptibility testing were performed via automated systems and standard biochemical methods following the Clinical and Laboratory Standards Institute (CLSI) guidelines applicable at the time.

Multidrug resistance was defined as non-susceptibility to at least one agent in three or more antimicrobial categories, following international consensus definitions [15]. Carbapenem resistance was defined as resistance to either imipenem and/or meropenem. Additional classifications included extended-spectrum β-lactamase (ESBL)-producing Enterobacterales, carbapenem-resistant Enterobacterales (CRE), and pan-resistant isolates.

Rectal swab cultures were obtained exclusively for antimicrobial resistance surveillance to identify colonization with MDR organisms, particularly VRE. These surveillance cultures were not considered diagnostic of infection and were therefore excluded from HAI incidence calculations and outcome analyses. Rectal swab findings are presented separately in tables as colonization/surveillance data, in order to describe resistance epidemiology without inflating the burden of true HAIs.

### 2.5. Statistical Analysis

Statistical analyses were conducted using IBM SPSS Statistics v26.0 (IBM Corp., Armonk, NY, USA). Continuous variables were assessed for normality (Kolmogorov–Smirnov test) and presented as the mean ± standard deviation (SD) or median (IQR). Categorical variables were summarized as numbers and percentages.

Group comparisons were performed using the χ^2^ or Fisher’s exact test for categorical variables and the Student’s *t*-test or Mann–Whitney U test for numerical variables, when appropriate. Receiver Operating Characteristic (ROC) curve analysis was used to determine optimal mortality prediction thresholds for CCI, SAPS II, and NLR. Multivariable logistic regression identified independent predictors of mortality. A *p*-value < 0.05 was considered statistically significant.

## 3. Results

During the nine-month study period, 1656 critically ill COVID-19 patients were treated in the ICU. Among these, 145 patients developed at least one HAI, yielding an overall HAI rate of 8.7%. After excluding nine patients due to incomplete clinical data, 136 patients were included in the final analysis.

### 3.1. Study Cohort and Baseline Characteristics

Baseline demographic characteristics and comorbidities across the three pandemic periods are summarized in Table 1. The mean patient age was 66.9 ± 13.6 years, with no significant differences among periods (*p* = 0.277).

Most patients were male (62.5%) and had at least one comorbidity (85.3%). The prevalence of diabetes mellitus and hypertension differed significantly across periods (*p* = 0.018 and *p* < 0.001, respectively), with both conditions most frequent during the third period. Chronic lung diseases (asthma/COPD) were significantly more prevalent in the second period (*p* = 0.011).

Both total hospital length of stay and ICU duration varied significantly over time (*p* = 0.0009). Patients admitted during the first pandemic period experienced the longest hospital stays (58.2 ± 36.0 days) and ICU stays (49.8 ± 32.6 days), with progressive shortening observed in subsequent periods.

### 3.2. Infection-Related Laboratory Parameters

Significant inter-period variation was observed in inflammatory markers (Table 2). CRP levels at ICU admission were higher in the second period compared with the first (*p* = 0.014), whereas peak CRP values were highest during the first period (*p* < 0.001). The timing of peak CRP occurred earlier in the second period (*p* = 0.017).

The highest WBC counts were recorded during the first period (*p* < 0.001), while the WBC peak occurred earlier in the second period (*p* = 0.010).

Admission NLR values were significantly lower in the first period than in the second and third periods (*p* = 0.005 and *p* = 0.0009, respectively). Similarly, PLR values were lower in the first period compared with the third (*p* = 0.0008). IL-6 concentrations also differed significantly across periods (*p* = 0.022), with the highest levels observed during the first period (*p* = 0.007 vs. third period).

Beyond single time-point comparisons, inflammatory markers demonstrated distinct temporal dynamics in relation to both infection timing and patient outcomes. Admission values alone showed limited prognostic discrimination, whereas peak and highest values more accurately reflected disease severity and infection-related inflammatory burden.

Correlation analysis revealed that admission NLR was negatively correlated with time to first microbiological growth, indicating earlier infection detection in patients with heightened inflammatory responses at ICU admission. In contrast, peak NLR and PLR values showed significant positive correlations with time to pathogen isolation, suggesting progressive inflammatory amplification in patients with prolonged ICU courses.

When stratified by outcome, non-survivors consistently exhibited higher peak inflammatory marker levels, particularly NLR and SAPS-II–adjusted inflammatory indices, compared with survivors. These findings support the concept that dynamic inflammatory trajectories, rather than isolated measurements, are more closely associated with infection emergence and mortality in critically ill patients with COVID-19.

Due to the retrospective study design and variability in laboratory sampling intervals, uniform longitudinal plotting was not feasible. Accordingly, inflammatory marker dynamics were interpreted using peak values and temporal correlations, and results were evaluated within these methodological constraints.

### 3.3. Mortality and Predictive Scores

Overall in-hospital mortality among patients with HAIs was 74.3% (n = 101), with the highest mortality observed during the third pandemic period. Non-survivors exhibited significantly higher MCCI, SAPS-II, SAPS-II (%), and peak NLR values (all *p* < 0.05). Interestingly, the MCCI percentile was significantly lower in non-survivors than survivors (*p* = 0.020), suggesting a nonlinear association between comorbidity burden and HAI-related mortality.

**Figure 1:**
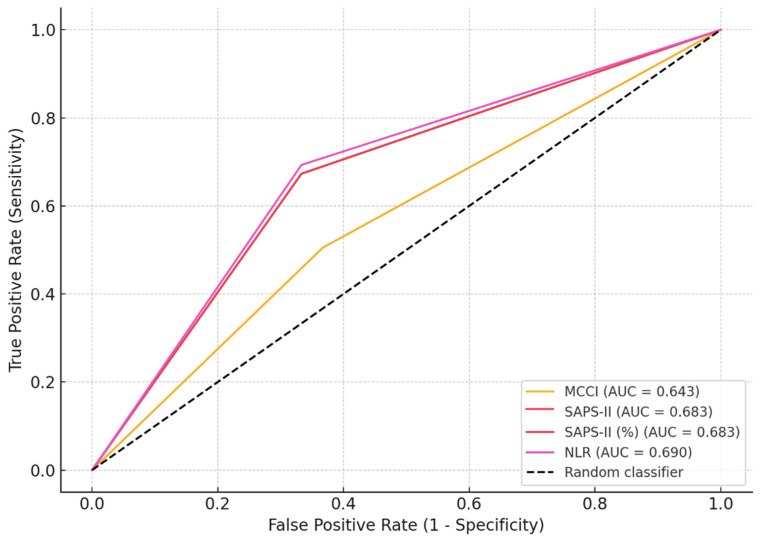
ROC Curve Analysis of Mortality Prediction in ICU patients with COVID-19-associated healthcare-associated infections.

ROC analyses identified optimal cut-off values for mortality prediction: MCCI ≥ 4.5 (AUC = 0.643, *p* = 0.017), SAPS-II ≥ 35.5 (AUC = 0.683, *p* = 0.02), SAPS-II (%) ≥ 17.4 (AUC = 0.683, *p* = 0.02), NLR ≥ 38.8 (AUC = 0.690, *p* = 0.02) and MCCI < 37 (AUC = 0.639, *p* = 0.02). These values indicate that inflammatory markers and severity scores were strong predictors of adverse outcomes (Table 2, Figure 1).

In multivariable logistic regression analysis, higher disease severity and inflammatory burden remained independently associated with in-hospital mortality (Table 3). SAPS-II score and peak NLR emerged as the strongest independent predictors of death, while comorbidity burden assessed by MCCI also showed a significant independent effect. Bloodstream infection was associated with increased mortality compared with other HAI types. In contrast, age, sex, multidrug-resistant pathogen status, and pandemic period did not retain independent significance after adjustment.

### 3.4. Pathogen Distribution

Time to initial culture positivity did not differ significantly across periods (*p* = 0.298). However, inflammatory marker dynamics were associated with pathogen emergence. Admission NLR showed a negative correlation with time to first microbiological growth (*p* = 0.045), whereas peak NLR and PLR demonstrated significant positive correlations (*p* = 0.0009 and *p* = 0.008, respectively).

A total of 373 microbial isolates were identified (Table 4). *Klebsiella pneumoniae* was the predominant pathogen (80.1%), followed by *Candida albicans* (37.8%) and *Acinetobacter baumannii* (36.8%). Significant inter-period variation was observed for several organisms. *C. albicans* and *Enterococcus faecium* were more frequent during the first period (*p* = 0.040 and *p* = 0.023, respectively). MRSA and *Pseudomonas aeruginosa* were also more prevalent in the first period compared with the second (*p* = 0.014 and *p* = 0.001, respectively). VRE colonization, identified exclusively through rectal swab surveillance cultures, was most frequently detected during the second period (*p* = 0.024) and was not included in HAI incidence calculations. Notably, *C. albicans* was significantly less common among non-survivors than survivors (*p* = 0.010).

### 3.5. Distribution of HAI Types

HAIs were dominated by bloodstream infections (BSIs), accounting for 57% of all episodes, followed by urinary tract infections (UTIs) (31%), ventilator-associated pneumonia (VAP) (9%), and surgical-site infection (SSI) (~1%) (Table 5).

BSIs were mainly associated with *K. pneumoniae*, *A. baumannii*, MRSA, and *C. albicans*. UTIs were commonly due to *K. pneumoniae* and *C. albicans*, followed by *E. faecium* and *C. tropicalis*. VAP cases most frequently involved *K. pneumoniae*, *A. baumannii*, and *P. aeruginosa*. SSIs were rare but predominantly caused by *K. pneumoniae*, MRSA, and *C. albicans*.

Detailed antimicrobial susceptibility profiles are provided in the Supplementary Materials.

## 4. Discussion

In this retrospective observational study of critically ill patients with laboratory-confirmed COVID-19, we evaluated the epidemiology of HAIs and their clinical determinants across three distinct pandemic periods. The overall HAI incidence of 8.7% observed in our ICU falls within the range reported in COVID-19 critical care populations, although it is slightly lower than pooled estimates from several international series [16,17,18]. Previous studies have reported HAI or superinfection rates of up to 40–50% among mechanically ventilated patients [9,19]. Such variability likely reflects differences in ICU burden, staffing ratios, infection prevention capacity, antimicrobial stewardship practices, and local pathogen ecology, particularly during surge conditions.

The high in-hospital mortality rate (74.3%) among patients with HAIs in our cohort underscores the substantial prognostic impact of secondary infections in severe COVID-19. This finding is consistent with prior evidence demonstrating that bacterial and fungal superinfections markedly increase mortality in patients already affected by virus-induced immune dysregulation [20,21]. BSIs, VAP, and catheter-related infections have been shown to exacerbate systemic inflammation, precipitate multiorgan failure, and prolong ICU stay [22,23]. In line with these observations, we found that patients with HAIs—particularly during the first pandemic period—experienced prolonged ICU and hospital stays, reflecting early uncertainties in treatment strategies, ventilation practices, and infection prevention protocols.

The temporal evolution of inflammatory and clinical parameters across pandemic periods provides important insight into the interaction between COVID-19 pathophysiology and nosocomial infection risk. Patients admitted during the earliest phase exhibited the highest levels of CRP, procalcitonin, WBC, and IL-6, consistent with the hyperinflammatory phenotype described before the widespread adoption of corticosteroids and immunomodulatory therapies [24]. This period was also characterized by longer ICU stays, greater reliance on invasive procedures, and increased exposure to device-related infection risk. As clinical experience accumulated and treatment protocols evolved, inflammatory burden and ICU length of stay progressively declined, paralleling reports of improved outcomes in later pandemic waves [25].

Severity-of-illness indices—particularly SAPS-II, MCCI, and NLR—were strongly associated with mortality in our cohort. Elevated NLR is a well-established marker of dysregulated immune response in COVID-19, and its prognostic significance has been consistently demonstrated in meta-analyses [26,27,28]. Our findings extend this evidence by showing that peak and maximum NLR values, rather than admission measurements alone, were most closely associated with both mortality and earlier microbiological documentation of infection. The inverse association observed between MCCI percentile and mortality suggests that acute disease severity and inflammatory burden outweighed baseline comorbidity in determining outcomes, an observation consistent with prior studies indicating that COVID-19-related organ dysfunction may supersede pre-existing frailty in critically ill patients [29].

From a microbiological perspective, the predominance of Gram-negative pathogens, particularly *Klebsiella pneumoniae* and *Acinetobacter baumannii*, mirrors global trends reported in COVID-19 ICUs. Earlier pandemic periods were characterized by higher frequencies of *Candida albicans*, *Enterococcus faecium*, MRSA, and *Pseudomonas aeruginosa*, suggesting dynamic ecological shifts driven by antimicrobial consumption patterns, immunosuppressive therapies, prolonged hospitalization, and strain on infection prevention systems [30,31,32]. These findings align with reports by O’Toole and Rawson et al., who highlighted pandemic-associated increases in multidrug-resistant organisms due to widespread empirical antibiotic use and weakened stewardship oversight [5,6].

Bloodstream infections constituted the majority of HAIs in our study, consistent with multicenter reports describing marked increases in catheter-associated bloodstream infections during pandemic peaks Prolonged mechanical ventilation, frequent prone positioning, repeated vascular access attempts, and staffing shortages likely contributed to this pattern. Early pandemic reliance on subclavian catheterization at our institution may also have increased BSI risk, as noted in similar tertiary-care settings [17,33].

The observed shift in fungal epidemiology, including a decline in *C. albicans* prevalence over time, may reflect improved antimicrobial stewardship, refined corticosteroid dosing, and strengthened infection control practices. The transient emergence of VRE colonization during the second period underscores the dynamic nature of hospital transmission during fluctuating ICU occupancy and highlights the importance of continuous microbiological surveillance.

Importantly, our results reinforce the interconnected relationship between systemic inflammation, invasive care, antimicrobial resistance, and patient outcomes. The significant correlations between NLR/PLR dynamics and time to pathogen isolation suggest that evolving inflammatory trajectories may predispose patients to earlier microbial proliferation—a concept previously proposed but rarely quantified in COVID-19-associated HAI research. The consistent dominance of *K. pneumoniae* across bloodstream, urinary, and respiratory infections further emphasizes the need for targeted empirical strategies in comparable ICU settings [5,8].

Several limitations warrant consideration. As a single-center retrospective study, our findings may not be fully generalizable to ICUs with differing patient populations, resistance patterns, or organizational structures. Antimicrobial consumption data were unavailable, although prescribing practices likely influenced resistance trends and pathogen distribution. The retrospective design precluded causal inference, and the absence of molecular typing prevented the assessment of clonal transmission or outbreak dynamics.

Additionally, due to incomplete availability of denominator data (e.g., ventilator days and central line–days), incidence density metrics could not be calculated, and HAI frequencies are presented as proportional distributions rather than standardized surveillance rates, limiting direct benchmarking with external datasets. Finally, evolving COVID-19 treatment strategies—including corticosteroids, IL-6 inhibitors, ventilation practices, and anticoagulation—may have independently influenced outcomes, complicating isolation of infection-specific mortality effects [12,25,34].

Despite these limitations, this study provides a longitudinal, wave-based analysis of HAIs in critically ill COVID-19 patients, integrating inflammatory biomarker dynamics, pathogen ecology, and severity scoring systems. Our findings highlight the persistent burden of HAIs, the central role of multidrug-resistant Gram-negative pathogens—particularly *K. pneumoniae* and *A. baumannii*—and the prognostic value of dynamic inflammatory markers. These observations underscore the need for adaptive infection control strategies, strengthened antimicrobial stewardship, and continuous microbiological surveillance to mitigate the impact of nosocomial infections during ongoing and future health-system crises.

## 5. Conclusions

In this retrospective observational study of critically ill patients with COVID-19, HAIs emerged as a major contributor to morbidity and mortality throughout the pandemic. BSIs, predominantly caused by multidrug-resistant *Klebsiella pneumoniae* and *Acinetobacter baumannii*, were the most frequent HAI type and were strongly associated with adverse clinical outcomes.

Severity-of-illness indices (SAPS-II and MCCI) and inflammation-based biomarkers (NLR, PLR, CRP, and IL-6) were closely linked to both the timing of infection onset and in-hospital mortality, highlighting their potential value for early risk stratification in critically ill patients. The observed temporal shifts in pathogen distribution and inflammatory profiles reflect evolving clinical practices, resource constraints, and antimicrobial exposure patterns across successive pandemic waves.

These findings underscore the need for adaptive infection-prevention strategies, continuous microbiological surveillance, and strengthened antimicrobial stewardship in ICUs managing patients with COVID-19. Future research should prioritize multicenter, prospective, and molecular-based approaches to better elucidate transmission dynamics, resistance evolution, and pathogen–host interactions. Despite its single-center design, this study provides valuable longitudinal insight into the evolving burden of HAIs during the COVID-19 pandemic and reinforces the importance of robust infection-control measures in protecting critically ill, immunologically vulnerable patient populations.

## Figures and Tables

**Table 1:** Baseline demographic and clinical characteristics across COVID-19 periods.

	Total	1st Period	2nd Period	3rd Period	*p*-Value
Age (years), mean ± SD	66.9 ± 13.6	65.9 ± 15.8	64.2 ± 14.5	69.6 ± 10.0	0.277
BMI (kg/m^2^), mean ± SD	29.5 ± 5.1	28.3 ± 5.8	30.2 ± 4.9	30.5 ± 4.4	0.328
Gender					0.681
Female	51 (37.5%)	19 (38.8%)	11 (31.4%)	21 (40.4%)	
Male	85 (62.5%)	30 (61.2%)	24 (68.6%)	31 (59.6%)	
Comorbidities					0.188
None	20 (14.7%)	9 (18.4%)	7 (20.0%)	4 (7.7%)	
Present	116 (85.3%)	40 (81.6%)	28 (80.0%)	48 (92.3%)	
Diabetes mellitus	56 (41.2%)	14 (28.6%)	13 (37.1%)	29 (55.8%)	0.018 *
Hypertension	76 (55.9%)	23 (46.9%)	13 (37.1%)	40 (76.9%)	<0.001 *
Coronary heart disease	36 (26.5%)	11 (22.4%)	9 (25.7%)	16 (30.8%)	0.634
Asthma/COPD	27 (19.9%)	6 (12.2%)	13 (37.1%)	8 (15.4%)	0.011 *
Cerebrovascular disease	14 (10.3%)	7 (14.3%)	4 (11.4%)	3 (5.8%)	0.359
Hydrocephalus	1 (0.7%)	0	0	1 (1.9%)	1.000
Hypothyroidism	10 (7.4%)	3 (6.1%)	4 (11.4%)	3 (5.8%)	0.641
Malignancy	10 (7.4%)	3 (6.1%)	2 (5.7%)	5 (9.6%)	0.766
Neurodegenerative	14 (10.3%)	8 (16.3%)	2 (5.7%)	4 (7.7%)	0.211
Chronic kidney disease	9 (6.6%)	2 (4.1%)	2 (5.7%)	5 (9.6%)	0.564
Tuberculosis	4 (2.9%)	2 (4.1%)	1 (2.9%)	1 (1.9%)	0.833
Prostatic hyperplasia	2 (1.5%)	2 (4.1%)	0	0	0.195
ASA score					0.486
ASA I	20 (14.7%)	9 (18.4%)	7 (20.0%)	4 (7.7%)	
ASA II	59 (43.4%)	20 (40.8%)	15 (42.9%)	24 (46.2%)	
ASA III	57 (41.9%)	20 (40.8%)	13 (37.1%)	24 (46.2%)	

Statistics: * *p* < 0.05. *p*-values from Chi-square test unless otherwise stated. Abbreviations: ASA, American Society of Anesthesiologists; BMI, body mass index; COPD, chronic obstructive pulmonary disease.

**Table 2:** Predictive Value of Admission Scoring Systems for ICU Mortality.

	Exitus (n = 101)	Survival (n = 30) **	*p*-Value
SOFA (before ICU)	3 (IQR 2–4)	3 (IQR 2–3.5)	0.798
SOFA (ICU)	5 (4–7)	4 (3–5.25)	0.093
MCCI	5 (3–6)	4 (2–5)	0.017 *
MCCI (%)	21 (2–77)	53 (21–90)	0.020 *
SAPS-II	40 (31–53)	31 (26.5–43)	0.002 *
SAPS-II (%)	24.7 (11.7–53)	11.7 (7.55–30.6)	0.002 *
IL-6 (first)	73.1 (27.5–313.2)	68.2 (23.6–302.9)	0.539
Procalcitonin (admission)	0.39 (0.175–1.45)	0.265 (0.18–1.42)	0.786
Peak PCT day	18 (9–28)	9.5 (5–24.25)	0.130
WBC (admission)	9.55 (6.13–13.1)	8.34 (5.90–12.85)	0.675
Highest WBC	31.33 (21.76–47.35)	31.79 (22.62–232.22)	0.509
NLR (highest)	50.2 (33.35–86)	36.4 (26.07–46.45)	0.002 *
PLR (highest)	796 (540–1282)	719 (559–976)	0.258

Statistics: * *p* < 0.05 statistically significant. Notes: ** Intubated patients transferred to another center were excluded. Abbreviations: ICU, intensive care unit; IL-6, interleukin-6; MCCI, Modified Charlson Comorbidity Index; NLR, neutrophil-to-lymphocyte ratio; PLR, platelet-to-lymphocyte ratio; SAPS-II, Simplified Acute Physiology Score; SOFA, Sepsis-related Organ Failure Assessment; WBC, white blood cell.

**Table 3:** Multivariable Logistic Regression Analysis of Factors Associated with In-Hospital Mortality in ICU COVID-19 Patients with Healthcare-Associated Infections.

Variable	Adjusted Odds Ratio (OR)	95% Confidence Interval	*p*-Value
Age (per 1-year increase)	1.02	0.99–1.05	0.148
Male sex	1.21	0.58–2.54	0.612
MCCI	1.18	1.03–1.36	0.018 *
SAPS-II score (per 1-point increase)	1.07	1.03–1.12	0.002 *
NLR, peak	1.04	1.01–1.07	0.006 *
Bloodstream infection (vs. other HAI types)	1.89	1.01–3.56	0.046 *
Multidrug-resistant pathogen	1.67	0.88–3.14	0.112
Pandemic period (3rd vs. 1st)	1.43	0.69–2.96	0.332

Statistics: Nagelkerke R^2^ = 0.41; Hosmer–Lemeshow goodness-of-fit test: *p* = 0.61; Overall model accuracy: 78.2%. * *p* < 0.05 statistically significant. Abbreviations: HAI, healthcare-associated infection; ICU, intensive care unit; MCCI, Modified Charlson Comorbidity Index; NLR, neutrophil-to-lymphocyte ratio; SAPS-II, Simplified Acute Physiology Score II.

**Table 4:** Distribution of isolated pathogens among ICU COVID-19 patients with healthcare-associated infections across the three study periods.

Pathogen	Total n (%)	1st Period n (%)	2nd Period n (%)	3rd Period n (%)	*p* #
*Klebsiella pneumoniae*	109 (80.15)	43 (87.76)	29 (82.86)	37 (71.15)	0.101
*Acinetobacter baumannii*	50 (36.76)	21 (42.86)	16 (45.71)	13 (25.00)	0.079
*Candida albicans*	50 (36.76)	25 (51.02)	10 (28.57)	15 (28.85)	0.035 *
MRSA	39 (28.68)	21 (42.86)	10 (28.57)	8 (15.38)	0.010 *
*Enterococcus faecium*	29 (21.32)	19 (38.78)	5 (14.29)	5 (9.62)	0.001 *
*Pseudomonas aeruginosa*	22 (16.18)	14 (28.57)	6 (17.14)	2 (3.85)	0.003 *
*Candida tropicalis*	17 (12.50)	5 (10.20)	5 (14.29)	7 (13.46)	0.826
*Candida glabrata*	12 (8.82)	3 (6.12)	4 (11.43)	5 (9.62)	0.696
*Candida parapsilosis*	9 (6.62)	6 (12.24)	1 (2.86)	2 (3.85)	0.231
*Escherichia coli*	6 (4.41)	2 (4.08)	1 (2.86)	3 (5.77)	0.876
*Stenotrophomonas maltophilia*	5 (3.68)	1 (2.04)	3 (8.57)	1 (1.92)	0.305
VRE (“Colonization”)	5 (3.68)	1 (2.04)	4 (11.43)	0 (0)	0.013 *
Carbapenem-resistant *Klebsiella*	4 (2.94)	2 (4.08)	1 (2.86)	1 (1.92)	0.836
*Providencia stuartii*	4 (2.94)	3 (6.12)	1 (2.86)	0 (0)	0.135
*Candida krusei*	3 (2.21)	2 (4.08)	0 (0)	1 (1.92)	0.621
*Burkholderia cepacia*	2 (1.47)	2 (4.08)	0 (0)	0 (0)	0.190
*Morganella morganii*	2 (1.47)	2 (4.08)	0 (0)	0 (0)	0.190
*Cryptococcus neoformans*	1 (0.74)	1 (2.04)	0 (0)	0 (0)	0.607
*Enterobacter cloacae*	1 (0.74)	0 (0)	0 (0)	1 (1.92)	1.000
*Klebsiella aerogenes*	1 (0.74)	0 (0)	0 (0)	1 (1.92)	1.000
*Serratia marcescens*	1 (0.74)	0 (0)	1 (2.86)	0 (0)	0.256

Notes: Total isolates: 373 (173 in Period 1; 97 in Period 2; 103 in Period 3). Statistics: # Chi-square test. * Statistically significant (*p* < 0.05). Footnote: VRE isolates were obtained exclusively from rectal swab surveillance cultures and represent colonization. These isolates were not considered healthcare-associated infections and were excluded from HAI incidence and outcome analyses. Abbreviations: MRSA: Methicillin-resistant *Staphylococcus aureus*; VRE: Vancomycin-resistant enterococci.

**Table 5:** Distribution of pathogens according to infection site among ICU COVID-19 patients with healthcare-associated infections.

Infection Site	*K. pneumoniae*	*A. baumannii*	*C. albicans*	*C. glabrata*	*E. faecium*	MRSA	*S. maltophilia*	*C. parapsilosis*	*C. tropicalis*	VRE	Others *
Blood culture	51 (46.8%)	28 (56%)	12 (24%)	1 (8.3%)	11 (37.9%)	22 (56.4%)	3 (60%)	6 (66.7%)	3 (17.6%)	0	14
Catheter-related bloodstream infection	15 (13.8%)	15 (30%)	10 (20%)	1 (8.3%)	1 (3.4%)	18 (46.2%)	3 (60%)	1 (11.1%)	0	3	10
Catheter tip	0	3 (6%)	3 (6%)	0	1 (3.4%)	7 (18.4%)	0	1 (11.1%)	0	0	2
Tracheal aspirate	17 (15.6%)	10 (20%)	1 (2%)	0	0	0	1 (20%)	0	0	0	9
Urine culture	39 (35.8%)	1 (2%)	30 (60%)	10 (83.3%)	15 (51.7%)	0	0	2 (22.2%)	15 (88.2%)	0	14
CSF	0	0	0	0	0	1 (2.6%)	0	0	0	0	0
Surgical Site Infection	4 (3.7%)	0	1 (2%)	0	0	0	0	0	0	0	0
Rectal swab #	0	0	0	0	2 (6.9%)	0	0	0	0	2 (100%)	0

Notes: Others * include: *E. coli*, *S. marcescens*, *E. cloacae*, *K. aerogenes*, *B. cepacia*, *M. morganii*, *C. krusei*, *C. neoformans*, CRK, etc. # Rectal swab isolates represent colonization and were not included in HAI incidence calculations [Colonization surveillance only]. Abbreviations: CSF: Cerebrospinal fluid; MRSA: Methicillin-resistant *Staphylococcus aureus*; VRE: Vancomycin-resistant *Enterococcus*.

## Data Availability

All data generated or analyzed during this study are included in this article. Further enquiries can be directed to the corresponding author.

## Supplementary Materials

The following supporting information can be downloaded at: https://www.mdpi.com/article/10.3390/medicina62010118/s1, Table S1: Antimicrobial susceptibility model of isolated pathogens. This table presents the antimicrobial susceptibility patterns of pathogens isolated from ICU patients with laboratory-confirmed COVID-19 who developed healthcare-associated infections. Values represent the number of susceptible isolates (*n*) and percentage of total isolates (%). Antifungal agents are shown at the top, followed by antibacterial agents grouped by drug classes.

## Abbreviations

ASA: American Society of Anesthesiologists physical status classification system; BMI: Body mass index; BSI: Bloodstream infection; COVID-19: Coronavirus disease 2019; COPD: chronic obstructive pulmonary disease; CRK: Carbapenem-resistant *Klebsiella*; CRP: C-reactive protein; DM: Diabetes Mellitus; HAI: Healthcare-associated infections; HT: Hypertension; ICU: Intensive care units; IL-6: Interleukin-6; MCCI: The modified Charlson Comorbidity Index; MDR: multidrug-resistant; MRSA: Methicillin-resistant *S. aureus*; NLR: Neutrophil to lymphocyte ratio; PLR: Platelet to lymphocyte ratio; PRC: Procalcitonin; SAPS-II: The Simplified Acute Physiology Score-II; SOFA: The Sepsis Related Organ Failure Assessment; SSI: Surgical site infections; UTI: Urinary tract infections; VAP: Ventilator-associated pneumonia; VRE: Vancomycin-resistant enterococci; WBC: White blood cell.
