# Supplementary material for: Healthcare-Associated Infections in Critically Ill COVID-19 Patients Across Evolving Pandemic Waves: A Retrospective ICU Study
Source: Medicina (Kaunas). 2026 Jan 6;62(1):118. doi: 10.3390/medicina62010118 (PMC12843191; doi:10.3390/medicina62010118)
Supplement: Supplementary file 1 [file medicina-62-00118-s001.zip › medicina-4053009-supplementary.pdf]

Supplementary Table S1. Antimicrobial susceptibility model of isolated pathogens
This table presents the antimicrobial susceptibility patterns of pathogens isolated from ICU patients with laboratory-confirmed COVID-19 who developed healthcare-associated infections. Values represent the number of susceptible isolates (*n*) and percentage of total isolates (%). Antifungal agents are shown at the top, followed by antibacterial agents grouped by drug classes.
Abbreviations: CRK: Carbapenem-resistant *Klebsiella pneumoniae*; VRE: Vancomycin-resistant *Enterococcus*; MRSA: Methicillin-resistant *Staphylococcus aureus*.

Antifungal Agents

Agent	*C. albicans* n (%)	*C. glabrata* n (%)	*C. parapsilosis* n (%)	*C. tropicalis* n (%)	*C. krusei* n (%)	*C. neoformans* n (%)
Amphotericin B	1 (2%)	1 (8.3%)	1 (11.1%)	0	0	0
Fluconazole	9 (18%)	0	1 (11.1%)	3 (17.6%)	0	0
Voriconazole	9 (18%)	0	4 (44.4%)	3 (17.6%)	0	0
Caspofungin	9 (18%)	1 (8.3%)	5 (55.6%)	3 (17.6%)	0	0
Micafungin	9 (18%)	1 (8.3%)	5 (55.6%)	3 (17.6%)	0	0
Anidulafungin	8 (16%)	0	4 (44.4%)	3 (17.6%)	0	0
Flucytosine	1 (2%)	1 (8.3%)	1 (11.1%)	0	0	0

Gram-Positive Bacteria

Agent	*E. faecalis* n (%)	MRSA n (%)	VRE n (%)
Penicillin	0	1 (2.6%)	0
Ampicillin/Aminoglycosides	5 (17.2%)	0	0
Vancomycin	21 (72.4%)	15 (39.5%)	1 (50%)
Teicoplanin	21 (72.4%)	12 (31.6%)	1 (50%)
Daptomycin	0	15 (39.5%)	1 (50%)
Linezolid	20 (69%)	15 (39.5%)	1 (50%)
Erythromycin	0	3 (7.9%)	0
Clindamycin	0	4 (10.5%)	0
Tetracycline	0	5 (13.2%)	1 (50%)
Streptomycin	2 (6.9%)	0	0

Gram-Negative Bacteria

Agent	*K. pneumoniae* n (%)	*A. baumannii* n (%)	*E. coli* n (%)	*P. aeruginosa* n (%)	*S. marcescens* n (%)	*E. cloacae* n (%)	CRK n (%)
Piperacillin–Tazobactam	1 (0.9%)	0	2 (33.3%)	0	0	0	0
Cefuroxime	0	0	1 (16.7%)	0	0	0	0
Ceftriaxone/Ceftazidime	1 (0.9%)	0	4 (66.7%)	7 (31.8%)	1 (100%)	1 (100%)	0
Cefepime	0	0	0	1 (100%)	0	0	0
Imipenem	11 (10.1%)	1 (2%)	2 (33.3%)	1 (100%)	0	0	0
Meropenem	9 (8.3%)	3 (6%)	2 (33.3%)	1 (100%)	2 (50%)	1 (50%)	2 (50%)
Ertapenem	2 (1.8%)	1 (2%)	2 (33.3%)	1 (100%)	2 (50%)	2 (50%)	0
Colistin	5 (4.6%)	11 (22%)	0	1 (4.5%)	1 (25%)	1 (25%)	0

Other Antimicrobials

Agent	*S. maltophilia* n (%)	*P. stuartii* n (%)	*B. cepacia* n (%)	*M. morganii* n (%)
TMP–SMX	3 (6%)	1 (4.5%)	1 (25%)	0
Ciprofloxacin	0	1 (13.6%)	0	0
Levofloxacin	0	1 (100%)	0	0
Tigecycline	0	5 (100%)	0	0
Gentamicin	0	9 (100%)	1 (5%)	0
Amikacin	0	2 (33.3%)	1 (100%)	0
Nitrofurantoin	0	4 (66.7%)	0	0

Pan-Resistance Summary

Pathogen	n	%
K. pneumoniae	56	51.4%
A. baumannii	15	3%
P. aeruginosa	5	22.7%
CRK	2	5%
